# Camouflaging in Developmental Language Disorder: The Views of Speech and Language Pathologists and Parents

**DOI:** 10.1177/15257401221120937

**Published:** 2022-09-19

**Authors:** Hannah M. Hobson, Annabel Lee

**Affiliations:** 1 University of York, Heslington, UK; 2 Hull York Medical School, Heslington, UK

**Keywords:** developmental language disorder, camouflaging, mental health, diagnosis

## Abstract

The term *camouflaging* describes behaviors that cover up neurodivergent difficulties. While researched in autism, camouflaging has received no systematic study in other conditions affecting communication, including developmental language disorder (DLD). This study explored camouflaging in DLD, drawing on the experience and expertise of speech and language pathologists and parents of children with DLD. Using a qualitative descriptive design, we interviewed six speech and language pathologists and six parents of children with DLD. The inductive thematic analysis considered three broad topic areas: What camouflaging behaviors do children with DLD do, the impacts of camouflaging, and what factors are associated with camouflaging. Camouflaging took a range of forms, with eight common presentations identified. Camouflaging reportedly delayed recognition of children’s language needs and affected interventions. Camouflaging reportedly impacted children’s exhaustion, mental health, self-esteem, personality, friendships, and how others view them. Research characterizing camouflaging in DLD could help reduce the underdetection of children’s language needs.

The term *camouflaging* refers to conscious or unconscious strategies or behaviors that minimize neurodiverse people’s neurodivergent characteristics, via masking and compensation (Hull et al., 2017). Such behaviors could be explicitly learned (e.g., forcing oneself to make eye contact after being told by others in the environment that it is polite and expected to do so), or developed implicitly (e.g., unconsciously mimicking the facial expressions of a conversational partner). Thus far, camouflaging has been the topic of study in the context of autism, but individuals with other neurodevelopmental conditions could also employ camouflaging, with potential impacts on detection, diagnosis, treatment, and outcomes.

In the study of autism, camouflaging has been argued to impact the detection of autism and negatively affect mental health outcomes. It has been discussed in the context of the female autism phenotype and suggested as a reason for the widespread under-detection of autistic women and girls (Hull et al., 2020). Nonetheless, camouflaging has also been argued to arise in autistic people of all genders, not just autistic women (Pearson & Rose, 2021). Camouflaging may mean that autistic individuals “pass” in social environments as nonautistic, but camouflaging appears to be detrimental to people’s mental health and wellbeing: Camouflaging is experienced as emotionally draining and leads to anxiety, stress, exhaustion, and confusion over one’s identity (Bargiela et al., 2016; Hull et al., 2017). Quantitative measures of camouflaging in autism have now been developed (Hull et al., 2019), which has begun to allow for the examination of what cognitive skills or problems predict camouflaging (Hull et al., 2021) and quantification of their association with mental health concerns such as suicidality (Cassidy et al., 2020).

Outside the study of autism, camouflaging has received little to no study in the context of other neurodevelopmental conditions, such as developmental language disorder (DLD). DLD is considered to affect around 7% of children (Norbury et al., 2016; Tomblin et al., 1997), but despite its common prevalence, it remains poorly understood and poorly known about by the general public (McGregor, 2020; Thordardottir & Topbaş, 2021). Appropriate detection of DLD is paramount for children to receive timely and appropriate support for their language needs and mitigate against potential negative outcomes, including poor academic performance, poor mental health, lowered self-esteem, and difficulties with social functioning (McGregor, 2020; Thomas et al., 2019).

There are numerous reasons to study camouflaging in DLD. If camouflaging behaviors mean children’s language needs go undetected by adults in their environment, describing camouflaging in DLD could help to highlight common behaviors that children with language needs display when masking a communication need. This could help in the development of measures for detecting previously undiagnosed DLD or other speech, language, and communication needs. Such research may also provide insight into how others perceive children and young people with language needs, especially when language needs are not disclosed or known about. Indeed, some scholars have argued that for young offenders, a population in which undetected language problems are common, unrecognized language needs may lead young offenders to appear rude, reticent, lazy, or uncooperative and may negatively impact the success of interventions and programs aimed at reducing reoffending behavior (Snow & Sanger, 2011). Recent experiments testing the perceptions naive adults make of young people with DLD suggest that not knowing that a person has DLD leads participants to judge an individual as less honest, less likable, and more blameworthy (Hobson, Woodley et al., 2022). In addition, given the emerging links between camouflaging and poor mental health outcomes in autism (Cassidy et al., 2020), the study of camouflaging in DLD could assist in understanding why this population is at greater risk of depression and anxiety (Conti-Ramsden & Botting, 2008).

The aim of this research was thus to provide a first exploration of the phenomenon of camouflaging in DLD. To do this, we sought the opinions and expertise of speech and language pathologists and parents of children with DLD. We aimed to explore (a) what behaviors do children with DLD employ to camouflage their needs; (b) what impact does camouflaging have on the detection and diagnosis of children’s needs, their therapy and support, and elsewhere; and (c) what factors parents and professionals felt increased camouflaging.

## Method

### Qualitative Descriptive Design

We used a qualitative descriptive design, an approach used in health research, particularly when researchers wish to describe phenomena that have not received research previously. Given that camouflaging has, to the best of our knowledge, not been systematically studied before in relation to DLD, we felt this approach would yield the most appropriate methodology for our current project. Descriptive qualitative studies examine the experiences of individuals, clustering together themes that are common to multiple individuals and can use multiple forms of data media, including qualitative interviews as used in the present study (Willis et al., 2016). This approach is “theory light” in that while approaches such as phenomenology or grounded theory are based on specific methodological and theoretical frameworks (Sandelowski, 2000), qualitative descriptive studies do not require alignment with a particular philosophical or epistemological stance. Qualitative descriptive studies are however based on the philosophical tenets of naturalistic inquiry (Lincoln & Guba, 1985). These designs are also analytically flexible and do not use pre-existing rules as per other approaches that have come from specific philosophical or epistemological stances. The approach to analysis is data-driven, representing an analytical method that remains “close to the data,” with surface-level themes and description at the level of the obvious (Graneheim & Lundman, 2004; Sandelowski, 2010), and can thus be described as inference-light, although still includes interpretative processes (Lambert & Lambert, 2012; Sandelowski, 2000, 2010).

### Participants

Six speech and language pathologists (SLPs) and six parents of children with diagnoses of DLD were interviewed for the present study. Our approach was to interview speech and language pathologists and parents, to provide breadth and depth to our analysis. It was reasoned that SLPs would have professional experience working with multiple children with DLD and could answer our interview questions drawing on their experiences with numerous cases. Parents, on the contrary, were likely to have deep experience with their specific child and could provide in-depth reports about their own child’s behaviors.

Our recruitment material stated that SLPs ought to have a minimum length of clinical experience of 1 year to take part (this was to ensure that SLPs’ views reflected sufficient clinical expertise working with children with DLD). The SLPs included in our study ranged in years of experience from 3.5 to 32 (Mean years of experience = 18.17) and worked in a variety of settings including pupil referral units (educational settings to which children who cannot be supported in mainstream education, commonly for behavioral reasons, are sent) and mainstream schools and resource bases (mainstream schools with specific additional support for certain special educational needs, e.g., language and communication needs). Parents included five mothers and one father. The children included three males and three females, with the age of DLD diagnosis ranging from 2.5 years to 10 years, and their present ages at the time of the interviews ranging from 6 years to 14 years. All participants were based in the United Kingdom or the Republic of Ireland. Our participants were recruited via social media, and via Engage with DLD, a recruitment platform that supports research on DLD. None of the participants were known to the research team prior to the study, nor to each other (i.e., the recruitment of families and clinicians was independent, and no SLPs referred parents to our study, or vice versa). Participants were compensated for their time with monetary vouchers.

### Procedure

Our study was reviewed and approved by our local ethics committee. All participants were informed of the aims of the study and completed online consent forms before the interviews.

We conducted semistructured interviews with our participants. To guide the conversations, we developed topic guides ahead of these interviews (as is recommended for qualitative descriptive designs; Willis et al., 2016). Our topic guides for our SLP, and parent interviews can be found in the online supplemental materials. Interviews took place over Zoom, and lasted between 45 min to 1 hr. All transcripts were anonymized, removing any names or places that could identify participants.

### Analytical Approach

Qualitative descriptive studies are analytically flexible and do not require a commitment to a given theory. Our own analysis followed the framework outlined by Willis et al. (2016) and was undertaken using NVivo 20. Analysis began with the transcription of interviews into written format (undertaken by AL), during which process data were suitably anonymized. HH then checked the accuracy of the final transcribed interviews. Transcribed interviews were then coded by the author team (initially by AL, with coding reviewed by HH), and codes organized into categories, using a thematic analytic approach.^1^ We took a largely inductive approach, given that this was a new topic of research, with limited background research to guide a deductive approach. Our aim was to keep themes “close to the data,” meaning that themes were not “going beyond or behind” the data but seeking to provide a valid surface-level description of participants’ reports (as outlined by Sandelowski, 2000). All final categories and themes were initially developed by AL, and reviewed and finalized by HH. The ultimate aim of qualitative descriptive analysis is to present a descriptive summary of the data, organized in a logical manner (Lambert & Lambert, 2012). Combining both insights from our SLP and parent participants, we arrived at a framework that we felt best captured the responses of all our participants. After developing this framework, interviews were reread to confirm final themes and subthemes reflected our participants’ experiences and views and confirm descriptive validity (Willis et al., 2016).

Given the qualitative nature of the study, we report here a brief positionality statement, to reflect the research team’s relationship to the topic of study: One author is an academic researcher, with a background primarily in psychology, and the other author is a medical student. Neither member of the author team are speech and language pathologists nor parents or family members of children or adults with language needs.

## Results

Following analyses, we derived three overarching themes that organized our findings: “What camouflaging behaviors do children with DLD do?” “The impacts of camouflaging” and “What factors are associated with camouflaging?” These are summarized in more detail subsequently. To support our analysis, online Supplemental Tables 1 to 3 include quotations from participants: Elements in bold in the sections below highlight key subthemes and connect to the structure of the Tables.

Interviews with parents began with questions about their experiences becoming aware of their children’s language problems and receiving a DLD diagnosis. This context is important for helping understand why children’s language problems may not have been initially recognized, either by families or by professionals, and how camouflaging may have contributed to this. As such, we provide a brief summary of parents’ diagnostic experiences, before reporting the main themes of camouflaging.

### Summary of Parents’ Experiences Receiving DLD Diagnosis

The six parents varied in terms of their experiences of when they had first become concerned about their child’s language, and in getting a diagnosis. Four of the six parents had initially been concerned when their child was very young (between ages 1 and 2 years), one had been concerned about her child’s early speech development but had thought the issue resolved until much later, and one family only became concerned when their child was around 7 years old (when it was apparent his language lagged behind that of his younger sibling and younger sibling’s peers). The wait between initial concerns and diagnosis of DLD was very long for some families: One parent was concerned about her daughter’s language development from 18 months, but her daughter was 10 years old when DLD was finally diagnosed. Two families had opted to have private speech and language assessments, due to long waiting times or the inability to get referrals for assessments via the publicly funded health services or the local educational authority. Three families (and their professionals) had wondered initially whether their child was autistic, or had dyslexia or attention-deficit/hyperactivity disorder, as opposed to DLD.

Parents’ experiences of schools’ understanding and response to their eventual DLD diagnosis were mixed: Some parents felt their school’s staff still did not understand the term and what it meant, some reported that while schools had support plans they could use for dyslexia or autism they did not have plans for DLD, and some waited several months after diagnosis for their school to put a special educational needs plan in place. Even where there has been understanding from school professionals, parents report difficulties accessing long-term speech and language support due to restricted resources at their schools.

### What Camouflaging Behaviors Do Children With DLD Do?

**Figure 1.**
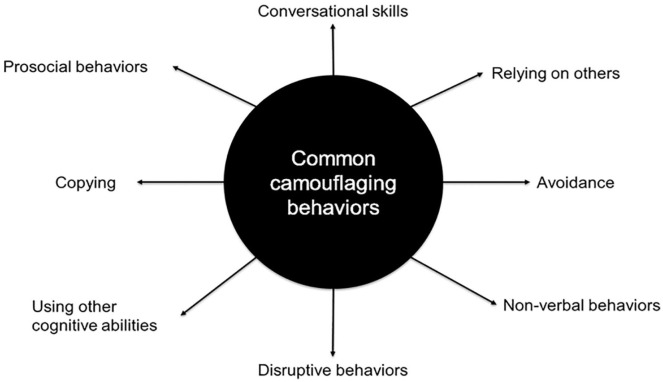
The eight common camouflaging behaviors described.

There was a wide variety of different camouflaging behaviors described by parents and speech and language pathologists. These are summarized in Figure 1. For examples of quotations that reflect these different presentations of camouflaging, see the online Supplemental Table 1.

It was reported that children used a variety of **conversational tools**, including phrases or tactics to keep a conversation going without people necessarily realizing they had not understood something or had expressive difficulties. One very simple tactic was to say that they had understood something when they had not (although it should be noted that some participants said they were not confident that all children with DLD had good awareness of when they had or had not understood something). Some children appear to have developed “scripts” around certain topic areas and might steer conversations to these topics, about which they had sufficient vocabulary and familiarity that their language might appear quite good (a characteristic that might contribute to a child appearing autistic in their profile). Conversational tools also included phrases to help bring a conversation to a close: These were used to politely finish an interaction, without drawing explicit attention to a child’s language problems (e.g., “I’m just tired today,” “Oh, I forgot.”)

The subtheme of **relying on others** referred to ways in which children relied on caregivers or adults in their environment to scaffold their interactions. Sometimes this scaffolding was elicited by children in quite sophisticated ways. For example, one participant described one child who appeared to behave in a way that was very young for his actual age; the result however was that adults in his environment spoke to him as if he was younger than he was, probably in a way that more accessible for him. Other ways that children utilized adults in their environment were by having conversational tools or techniques that invited help, for example, pausing and allowing people to fill in with words they might not know or have trouble accessing.

For some children, their camouflaging behaviors included **avoidance** of environments that relied on their language skills. This could include opting to play by themselves, choosing activities that did not rely heavily on language, or even physically removing themselves during an interaction. Some children befriended younger children, whose language skills were closer to their own, or they hung around adults at school, who were perhaps more accommodating than their same-age peers. Participants noted that some children were very quick to say they “don’t know,” or would pretend that they found the task in front of them too easy (and thus beneath them), to avoid doing it.

Some children employed **prosocial behavior** to gain likeability with peers and adults in their environment. In addition to smiling and nodding (also a feature of the nonverbal behaviors described below), children would display behaviors that were in line with being a “good student.” For example, children might always put their hand up in class, despite never getting the right answer. However, participants felt that such behavior would be perceived by a teacher to show sufficient evidence of engagement and motivation to participate. Other prosocial tactics included simply always agreeing with people and trying to use humor (although sometimes attempts at humor overlapped with disruptive, giddy or silly behavior, described).

**Nonverbal camouflaging behaviors** included smiling and nodding. Some participants described children’s smiles cues as forced, even like a grimace. Other nonverbal behaviors included uses of gesture, such as pointing. Finally, some nonverbal behaviors bought children time to process the language around them: One SLP had detected that one of the children she worked with often had their phone out, but it acted as something to look at and use to delay a response to a question (although the SLP noted that of course to many adults this behavior would come across as rude or disengaged).

Some children appeared to engage in “silly” behavior, becoming giddy and even laughing. For some children, acting out behavior tipped into what could be described as **disruptive behavior**. These behaviors might serve to try and change the progress of a conversation; giddy or silly behavior might be an attempt to stop a conversation or change the course of an interaction. More extreme disruptive behaviors could also be considered a form of avoidance (e.g., storming out during an assessment to avoid being made to complete it).

Some camouflaging behaviors made use of children’s **other cognitive abilities**. Children might be able to decode when reading in class, which might disguise a language need. Children would also make use of visual cues in their environment to help them know what to do. Children’s social abilities were sometimes noted as a potential strength: This allowed children to get help from friends. Some parents felt their children were good problem solvers and would work around a language problem to get their point across.

Another camouflaging behavior (arguably representing a particular cognitive skill, but one that was common enough in our interviews it was felt it deserved its own category) was children employing **copying**: Children could pick up the likely behavior or activity they were supposed to be doing by watching their peers around them and do what they were doing. In some cases, being caught copying got children into trouble.

### The Impact of Camouflaging

In addition to describing the nature of camouflaging, we asked parents and SLPs about the potential impacts of camouflaging. In our analyses, we distinguished impacts on clinical care (i.e., diagnosis of DLD and support for DLD) and impacts on other aspects of the children’s daily lives, specifically their personality and friendships, others’ perceptions of them, and their exhaustion, mental health, and self-esteem. Example quotes can be found in online Supplemental Table 2.

There was a potential impact on **diagnosis** and **treatment** for children with DLD. Successful camouflaging was thought to delay referrals to SLP services. In addition, camouflaging impacted the success of supports put in place. For instance, SLPs reported coming up with strategies that children could use to communicate with their teachers (e.g., picture books) or notify teachers that they were not understanding something (e.g., putting up colored flashcards in class). However, children were quick to stop using such techniques because they felt uncomfortable being different from other children, preferring to pretend to have understood instructions. Some SLPs reported children camouflaging even within speech and language therapy sessions; indeed, moving sessions to an online environment during the pandemic afforded new possibilities for children to camouflage, as one report of a child Googling answers to a vocabulary assessment portrayed. This example also highlights that children are motivated to minimize the appearance of their language needs, even when working with SLPs, adults who it might be assumed children would feel safer showing their communication needs to.

Aside from the impact on clinical care, there were other broader impacts on children, too. There were concerns about the impact of camouflaging on children’s **exhaustion, mental health, and self-esteem**. It was clear from interviews with parents that camouflaging was very tiring for children and that after school children would be emotional or irritable, and easily upset by their parents. It was felt that camouflaging could affect the development of good self-esteem and a sense of self if camouflaging behaviors meant ridiculing oneself or prevented children being able to share their authentic selves with others. Some parents reported that their children did not want to go to school or would adopt a different persona when at school. Other parents expressed concerns that as their children got older their language problems would **impact their personality and their friendships**: One parent described her daughter as a “social butterfly” but that over the last year she had become more withdrawn. One SLP described a teenage boy who appeared to a “strong and silent” member of his social group: In reality, this was not his true personality, but he found it very hard to keep up with the jokes and banter of the group. He found it easier to put on a persona of a deadpan individual, rather than laugh at the wrong places. Finally, there were concerns about how camouflaging impacts **others’ perceptions of their child’s personality and cognitive ability**. Some parents felt their children were perceived as “difficult” or rude, especially if children used avoidance or disruptive behaviors to try and exit or change the course of an interaction. SLPs also reported that sometimes other staff felt children were pretending not to understand things in SLP assessments. SLPs expressed that they felt educational staff did not always believe the child needed the support they were outlining.

We were mindful of asking participants about any potential positives to camouflaging. These were generally overlapping with children’s motivations for camouflaging: that they may be less likely to get into trouble for not having listened, that it may allow them to fit in with a social group, and that it may protect children from feeling that others think they are stupid. Indeed, one SLP made the point that in some circumstances having other people know a young person had a learning disability could leave that young person more, not less, vulnerable. However, participants felt these positives may be relatively short-term, and were outweighed by the negative impacts of camouflaging.

### What Factors Are Associated With Camouflaging?

Participants were asked if there was anything they felt increased the likelihood of children camouflaging or affected the presentation of camouflaging. Some factors were concerned with individual differences in the children themselves: These were children’s **personality** and their **cognitive abilities.** Participants also outlined several potential reasons that might increase children’s **motivation to camouflage**. In addition, participants noted some **environmental factors** that were felt to increase the likelihood of camouflaging. Example quotes relating to these factors are summarized in online Supplemental Table 3.

Specifically, with regard to **personality**, some participants said they felt children who were socially motivated would be more likely to camouflage (this links to some of the motivations put forward for children to camouflage, described below). Children’s personalities also influenced what sorts of camouflaging behavior they might adopt: Children who did not want to draw attention to themselves might adopt more passive camouflaging behaviors (e.g., avoidance behaviors), whereas children with more outgoing personalities might adopt different behaviors (e.g., giddy or disruptive behaviors).

With regard to **cognitive ability**, SLPs suggested that children who had cognitive strengths in other areas might utilize these to camouflage. Some professionals even suggested specific skills that might help, such as children being good readers being able to use this to appear to keep up in class. The notion that children with certain cognitive strengths might be more likely to engage in camouflaging holds clear links to the “using other cognitive abilities,” discussed previously.

Parents and speech and language pathologists suggested various reasons why a child might be **motivated to camouflage**. As noted earlier, this included camouflaging for social reasons (i.e., wanting to be liked or thought well of by peers). Other motivating reasons included that people knowing about their communication problems might make them feel vulnerable and thus camouflaging provided a sense of safety. For some children, it was felt that they camouflaged because they did not want to get into trouble: In classroom environments, not understanding might be interpreted as children not paying attention or not listening, and children might be told off for this. Camouflaging was also suggested to make daily interactions feel easier for the children: They could get through a conversational exchange with camouflaging rather than having to go through the effort of having to unpack what they had not understood every time. Finally, some suggested that camouflaging had been reinforced in children: because the consequences of camouflaging in the immediate term might be being liked, not getting into trouble, making things easier, and so on, these behaviors become reinforced. Over time, this could lead to children camouflaging largely unconsciously or without explicit motivation for doing so.

While children’s personalities, cognitive abilities, and motivations could be considered child-related factors that may increase the likelihood of camouflaging, there were however also **environmental factors** that were suggested to increase camouflaging. Parents reported that children were less likely to camouflage at home. SLPs observed that some children seemed to behave differently in small group activities versus typical classes. These environmental factors likely reflect children’s motivations for camouflaging: In smaller groups or with highly familiar people, they may feel safer, less likely to be punished for not understanding, and already accepted rather than needing to work to be liked.

## Discussion

The present study aimed to explore the topic of camouflaging in DLD, specifically investigating the presentations of camouflaging in DLD, the impact of camouflaging on children, and what factors may be associated with camouflaging. Our analyses of interviews with parents and SLPs highlight that camouflaging is very varied in DLD: Eight particular presentations were distilled, but we would argue this is unlikely to be an exhaustive list, rather a list of particularly common behaviors. Our findings also highlighted the potential impact camouflaging had on children, both on their clinical care but also on their social and well-being outcomes. In terms of what factors participants felt increased the likelihood of camouflaging in DLD, SLPs noted cognitive ability and social motivation as important, and personality was thought to impact what kinds of camouflaging behaviors were used. Children appeared more likely to camouflage away from the home and trusted individuals.

There are some notable similarities and differences between our present findings and the literature on camouflaging in autism. This initial study does not seem to suggest a strong link to gender, contrary to what has been discussed in the context of autism, where there has been the suggestion that camouflaging may be particularly prevalent in autistic girls (although there has been some disagreement regarding the linking of camouflaging to gender in autism; see Pearson & Rose, 2021). SLPs did not generally report that girls were more likely to camouflage, and camouflaging was reported by the parents of both boy and girl children in our sample. However, in agreement with research on camouflaging in autism, camouflaging does appear to be linked to exhaustion (see Harmens et al., 2022, for consideration of exhaustion in the experiences of autistic women). Indeed, reports of camouflaging in autism have noted problems with a sense of self, where constantly pretending to be someone else is experienced as disruptive to knowing one’s own identity (Bargiela et al., 2016). These appear to be echoed by the concerns of the SLPs and parents in our own study, with reports of children who did not feel they were their authentic selves, or where parents felt their children were putting on a positive persona when they were not, in truth, happy at school. In addition, as per the literature on young offenders, and speculation that young offenders with language needs may be perceived as uncooperative or rude (Snow & Sanger, 2011), our participants did note concerns about how children and young people with DLD were perceived, with some reporting experiences where colleagues in education doubted that children “needed” support.

Our findings should be considered a first examination of camouflaging in DLD, and further research will be needed before any large-scale changes in clinical practice. Nonetheless, we posit some implications for clinicians. First, we believe that speech and language professionals working with colleagues in education, for example, children’s teachers or teaching assistants, may find it useful to actively bring up the potential of camouflaging in such cases. For support to be implemented successfully, it is important that staff are in agreement that it is needed. Teachers or teaching assistants that do not agree with the need for support, or who underestimate a child’s language needs, may not carry out suggested support plans as desired. Indeed, researchers have argued for more systematic research into treatment fidelity when studying interventions for language (Haring Biel et al., 2020), but the available evidence does suggest that it may be a problem: In the Sound Start Study, a program of research on interventions for speech sound disorders, less than one-third of children received the prescribed number of days of intervention (McCormack et al., 2017). Poor fidelity could be due to a number of reasons, but ensuring that staff delivering support agree that children need the support they are giving could help ensure interventions remain a staff priority and have buy-in from educational professionals.

Second, while the SLPs in our study did mention the mental health impact of camouflaging, these reports were particularly powerful from the parents, and parents noted that they felt their children “held it together” while at school but were exhausted and had meltdowns when at home. This has implications for what clinicians and educational professionals get to see of children’s behavior and may mean that reports from school staff versus parents about children’s mental health and behavior do not agree.

Finally, we posit that explicitly considering camouflaging in speech-language therapy could be helpful for some children and young people. Many of our participants were unsure that children with DLD were that aware of their camouflaging behaviors. Indeed, for some, it was felt certain behaviors had become highly ingrained and were likely being done with little deliberate planning. Bringing camouflaging to the attention of children may help them notice these behaviors and allow them better control over their behavior. We would be reluctant at this stage to say that children should, at all times, be entirely discouraged from camouflaging. For example, for some children camouflaging may afford them access to social groups, bringing a sense of belonging. In addition, while some camouflaging behaviors may be especially problematic, some might actually be better than others at supporting children and young people with DLD to be included: For example, research has suggested that prosocial behavior is a protective factor for social outcomes for young adults with DLD (Toseeb et al., 2017). Further research will be needed to fully understand whether camouflaging has a net negative effect on young people and the nuances of different camouflaging strategy effects. However, consideration of what might happen if someone does not understand you have misunderstood, and working through what situations it might be best to choose not to camouflage, could support children to feel more comfortable to be themselves, ask for help, and relieve the burden of camouflaging effort, at least for some parts of the day.

With regard to future directions for research, there are a number of limitations in the current study and the available evidence base that future research would do well to address. First, we did not interview the young people themselves, and gaining their perspectives will be vital for understanding why children and young people with DLD camouflage. If research confirms our initial findings that camouflaging is largely a negative predictor of children’s outcomes, and clinicians wish to develop interventions to prevent or stop camouflaging, it will be necessary to understand the motivations children and young people have for camouflaging, to persuade them to stop.

Second, it would be worth exploring the utility of developing checklists or screening measures that could help identify and even quantify camouflaging in DLD. As the current project was, to our knowledge, the first to explore the topic of camouflaging in DLD, we opted for a qualitative descriptive approach, but the initial findings generated from our analysis could be used to create pilot quantitative measures. Indeed, self-report measures of camouflaging have been developed in the field of autism (Hull et al., 2019). For clinical purposes, a well-developed measure of camouflaging for DLD could help detect unrecognized language needs. It could also help characterize children coming into speech and language therapy: A child who camouflages a great deal may benefit from sessions that consider this issue specifically, and clinicians may need to be mindful of feeding back to educational staff that communication needs may be well hidden behind a range of behaviors. A challenge to developing such tools will be the varied nature of camouflaging, which seems apparent from the data we have collected so far: If the behaviors children engage in to disguise their communication needs are so varied, even to the point of being idiosyncratic, developing a standard measure will be very challenging. Quantitative measures may help detect common camouflaging behaviors but may not identify camouflaging in all children.

Third, assuming quantitative measures of camouflaging could be developed, the present article posits several initial suggestions for what factors might predict camouflaging in DLD. For research purposes, these measures can help to demonstrate the strength of associations between camouflaging behaviors and mental health outcomes or predictor variables. For example, in the field of autism, research with the Camouflaging Autistic Traits Questionnaire (CAT-Q) has highlighted links to suicidality (Cassidy et al., 2020) and illuminated the relationship between camouflaging and executive function (Hull et al., 2021). Parents and professionals highlighted the roles of cognitive ability, personality, motivating factors, and environmental factors in the likelihood of a child camouflaging. Future research should look to examine with quantitative methods the predictive power of such factors in understanding children’s camouflaging behaviors.

In addition, camouflaging could be a useful construct for understanding some of the known common difficulties experienced by children with DLD. First, camouflaging could add to our understanding of the social difficulties faced by children with DLD. The social adaptation model posits that the poor language skills of children with DLD lead peers to develop more negative opinions of them, leading to social exclusion, which then compounds these children’s social difficulties further by reducing the opportunities children have to develop social and communication skills with peers (Redmond & Rice, 1998). If we consider camouflaging in this model, it provides a clear rationale for children to camouflage, to avoid being ostracized. Children with DLD are also at an elevated risk of anxiety and depression (Conti-Ramsden & Botting, 2008; and indeed, social functioning might be especially relevant to the development of emotional problems; see Forrest et al., 2021), many parents of children with language needs express concerns about their children’s mental health (Hobson, Kalsi et al., 2022), and there is a high rate of unrecognized language problems in children with emotional/behavioral problems (Hollo et al., 2014). Our findings would suggest that camouflaging contributes to mental health problems, via exhaustion, degraded self-esteem, and a disrupted sense of self, but we posit that it may also contribute to the relative low detection of children with language needs in mental health services.

Of course, a key limitation in our study is that the data from parents and SLPs are related to children who have been diagnosed with DLD. However, highly successful camouflagers may not ever receive a diagnosis, as their behaviors may prevent such children ever being referred to a speech and language professional. Nonetheless, these individuals would of course be important to study and identify, to offer support.

### Conclusion

To summarize, the present study highlights that children with DLD engage in camouflaging behaviors that disguise their communication needs. These behaviors are varied and include conversational tools, avoidance behaviors, prosocial behaviors, and copying behaviors (and more). The impacts of camouflaging were considered largely negative in the long term, including impacts on diagnosis and interventions, and parents reported high levels of exhaustion in their children. There were concerns that camouflaging affects children’s sense of self and also the perceptions of those around them. The varied nature of camouflaging will pose a challenge to future research wanting to develop measures of camouflaging for use with this population. Nonetheless, future research in camouflaging could help identify children with language needs earlier and support children to have better mental health and social outcomes.

## Supplemental Material

sj-docx-1-cdq-10.1177_15257401221120937 – Supplemental material for Camouflaging in Developmental Language Disorder: The Views of Speech and Language Pathologists and Parents Click here for additional data file. Supplemental material, sj-docx-1-cdq-10.1177_15257401221120937 for Camouflaging in Developmental Language Disorder: The Views of Speech and Language Pathologists and Parents by Hannah M. Hobson and Annabel Lee in Communication Disorders Quarterly

## Notes

1. This is *not* the widely used, but also widely misunderstood, method of the reflexive thematic analytic method used by Braun and Clarke (2006); the reflexive thematic analytic method requires the development of deep reflexive themes rather than domain categories, and while an excellent analytical framework for the right question, we did not feel it was suitable for a study with the aim of largely *describing* a phenomenon.
