# Supplementary material for: Camouflaging in Developmental Language Disorder: The Views of Speech and Language Pathologists and Parents
Source: Commun Disord Q. 2022 Sep 19;44(4):247–56. doi: 10.1177/15257401221120937 (PMC10354791; doi:10.1177/15257401221120937)
Supplement: sj-docx-1-cdq-10.1177_15257401221120937 – Supplemental material for Camouflaging in Developmental Language Disorder: The Views of Speech and Language Pathologists and Parents [file sj-docx-1-cdq-10.1177_15257401221120937.docx]

**Supplementary Materials**

| ***Supplementary Materials Table 1: Example quotes for subthemes of “What camouflaging behaviors do children with DLD do?”*** | |
| --- | --- |
| Subtheme | Quotes |
| Conversational tools | He does football and the coach will say things like “[CHILD], can you please use more precision?” and he’ll just go “Yep”. And he hasn’t got a clue what they’re talking about, you know. – Parent 1  I’ve noticed that with conversations with people she sort of tends to steer it to something that she’s confident talking about. – Parent 5  He’s University age. As he’s talking, when you actually stop and analyse what he’s saying, I don’t think there’s one sentence that he manages to finish off. It’s kinda “ums “and “errs”, but also all these kind of socially acceptable phrases, which just kind of wraps the sentences up, because he couldn’t think of how to finish off. – SLT 4 |
| Relying on others | We’re providing him with the context, often, and the same thing I think happens with his friendship groups as well. – Parent 1  If I just like repeated back the first thing you said- then what you would naturally do is show me or say it again – you’re just giving people cues that you need more information on a kind of passive level. – SLT1 |
| Avoidance | She gets on quite well with boys because I think boys in the playground tend to less verbal and more active, so she would often do a lot of running around games. She would sort of divert it to that. – Parent 3  That learnt behavior of just getting up and walking away and making a fuss because I can, and yeah that means people leave me alone and I won’t have to do this horrible work again. – SLT 3  School refusal, or not wanting to go on trips, or different things like that. – SLT2 |
| Pro-social behaviors | Maybe he’s just learnt if you agree people just kind of leave you alone, you don’t have to say much else… – SLT3  You can see the fact that he is able to smile and look like he knows what he’s been asked to do. – Parent 2  She’s very like, sociable. She’s very chatty so she gets away with it sometimes, yeah. – Parent 4 |
| Non-verbal behaviors | If you just smile and laugh or just smile and nod, obviously you don’t have to say anything but also, people are never concerned about you. You blend into the background. – SLT 1  He will nod and say yes, smile and look like he knows. – Parent 2  …just pointing a lot and just trying to get away with not saying much at all but using a lot of gesture. – SLT 2  I think she puts a big smile on her face, my daughter. She’s very good at it. And I think you know a lot of people look at the smile and think, oh well, she’s happy, you know it sort of gets slightly lost, doesn’t it? – Parent 5 |
| Disruptive behavior | ‘Ugh’ [imitates child tossing head] Like refusing to engage because it’s like ‘Oh, this is really stupid. I can already do this kind of thing’, but obviously they can’t do it. – SLT1  Refusing and opting out are two terms I hear a lot. – SLT6  Being sort of giddy and laughing a lot and joking a lot, you know. I can think of one girl that used to use humour…she was just very giddy, is the best way to describe it. – SLT2 |
| Using other cognitive abilities | She’s looking round the room for information that she needs. She's obviously looking at what neighbours are doing, copying what others are doing...I suppose she's got really good copying skills and her handwriting is neat. And sometimes I think they are things that some teachers look for, don't they? But I think that helps her to sort of go under that radar. – Parent 5  So they're reading - you know they can read, they can decode the word and read, but then when you ask them questions, they don't actually know what they're reading. So maybe those ones that we have quite strong literacy skills in that sense. - SLT 3 |
| Copying | She came home once and said she got told off for copying somebody’s work. – Parent 5  He’s not following the coach’s instructions. He is very much ‘sit back and watch what the other kids are doing,' so he’s mimicking the other kids, he’s getting the cue from them… - Parent 6 |

| ***Supplementary Materials Table 2: Example quotes for subthemes of “The impact of camouflaging”*** | |
| --- | --- |
| Subtheme | Quote |
| Diagnosis | I think that the masking behaviour does really, really make a massive a difference in trying to make a diagnosis in children who are particularly motivated [to camouflage] on whatever level of consciousness – SLT 1  And I think there's a danger that children that camouflage like that perhaps don’t get picked up even as quickly as the ones that have the challenging behaviour because I think teachers think they are okay, and to leave them to get on. And this is a boy in year six as well that I'm thinking of that’s being very quiet and quite withdrawn. And when I work with him, he's got quite significant language disorder. -SLT 6  They thought it might be ASD, so we went down the ASD route and ADHD, and had all these kind of other diagnoses and no one could really do it, and so it’s really that just constant reading and searching and looking for an answer. -Parent 3 |
| Treatment | I’ve definitely had a lot of students Googling answers during their therapy and they think that I don’t know they’re typing but I can hear them. – SLT3  They will sign at home with their families, but they’re not so keen on signing when they’re in their nursery settings. Similarly with visual support, so we use a lot of visual passports, using symbols for children with DLD to be able to communicate…And there are children who will use symbols comfortably at home, but are less comfortable using them with peers because they don’t want to look any different. – SLT4  We’ve had trouble doing speech and language after school because she’s so tired. – Parent 5 |
| Exhaustion, mental health and self-esteem | Having to maintain that type of camouflage in school does take its toll and he does get very tired. – Parent 1  She gets home and she literally just can’t keep it all in and the smallest thing, she literally has a meltdown, but I think she’s finding it really hard to keep up and try and follow everything. – Parent 5  She comes home and she's so tired. And then she can't go to sleep because she can't switch off. But then in the morning, she can't get up because she's too tired. So, it's like it's a double-edged sword and it impacts so much on everything. -Parent 4  She wants constant reassuring and constant praise for the things that she does right. So if you say to [CHILD] that “that sentence doesn't read correctly, can you go back?”, she would say something like “Could you not say that in a nice way? Could you not say that the first 2 words are good and the rest is bad?” – Parent 3  With her, it's like the Coca Cola bottle effect - you describe it in autism - you know like that you know she'll be fizzing up, fizzing up, fizzing up all day and then once she gets home it's like brrrrr [mimics mini explosion]. - Parent 4  That's not healthy, you know, for a small child to be constantly laughing at themselves and making a joke of themselves. That’s something that people develop when they have more self-awareness and their, you know, their ego is more intact, not when they're so small. – SLT 1 |
| Impact on personality and friendships | He will openly say ‘I like talking to adults. I don’t like talking to other boys in my class’, and I think that’s because the adults are making accommodations for him and bridging that gap with his understanding’. – SLT6  He’s an incredibly serious guy, his personality is very serious, and he said, you know, it couldn’t be further from the truth. ‘I love humour. I love having a joke and I’m a very outgoing, fun loving person. But when I am in a group of friends, the banter is so quick, that I can’t process everything that’s going on… So the way that I cope with that and mask those problems is just to remain silent. And because I don’t laugh at the right places I come across as being incredibly serious’. – SLT4  But then as soon as she got in the porch way of school, there were- it was like a different child. It was like this different persona switched on, like a robot. And she just walked in ‘hiya [TEACHING ASSISTANT NAME]’ and walked straight into her class… Now, I’ve had to like fight for her to get ready. Fight for her to put on her shoes. Fight to get her in the car, fight to get her out the car, but as soon as she got to that porch way, it was like a different kid. If she [the teaching assistant] hadn't seen that she had red eyes, she’d have thought I were- there was something wrong with me. – Parent 4  What’s that doing to the child’s mental health is the other issue - if you're constantly not able to share who you are, who you truly are? They don't know anything about you. They don't ask about you. You can't share that you've got worries on your mind. Neither can you share that you've just got a new puppy, and you know you're really excited, and you’re going to your grandparents at the weekend - so, that sense of who I am. – SLT 4 |
| Others’ perceptions of their child | [I explain to the teachers:] She will need keywords written down, overlearning of vocabulary, you need to reduce your language, check that she’s understood – and people have to believe they need that. And the camouflage can hide the fact that they need it. – SLT2  I would get an assistant to sit with me whilst I did a test, so they can see. And you’ll have them say “she messing you about”… they won’t often say that in the session but afterwards they’ll say “I think she was just having you on” – SLT2  She also like interrupts a lot and that can come across as rude with people, but it’s not. It’s because if she doesn’t get out what she has got on the tip of her tongue, she’ll forget and she might not be able to get out what she needs to say. – Parent 4  I think a lot of people think CHILD is quite crazy – Parent 5 |

| ***Supplementary Materials Table 3: Example quotes for subthemes of “What factors are associated with camouflaging?”*** | |
| --- | --- |
| Subtheme | Quote |
| Personality | I think most if it is personality, you know. If you've got a full on active young guy that's very energetic, he's not going to be the child that sits quietly in the classroom with it going over his head, not doing anything. He's more likely to be the child that perhaps plays up a bit. – SLT5  Perhaps a bit more shy, weighing the situation up, a bit more anxious compared to the child who’s a lot more boisterous, impulsive, just barges in there a bit more often rather than trying to disguise it… - SLT 4 |
| Cognitive ability | I think it's not common in my experience to see children with really significant cognitive difficulties on a global level, masking that effectively. – SLT1  Children with much lower cognitive ability might not have this social empathy, to sort of think about and appreciate that, but these children want to fit in and appear like they don’t have difficulties… – SLT2 |
| Motivation | They want to please, you know. They want to say and do the right thing. – SLT 2  I think he didn’t want to get into trouble. That was his motivating factor, and he wanted social contact but didn’t have the language to get it. And that was his way of hiding it. – SLT 6  She fights every day to be like her peers. She just wants to do what they’re doing. She wants to be like them… so she’s had to develop this persona of being like everyone. – Parent 4 |
| Environmental factors | I think perhaps when he is more unsure, the more he'll do it. – Parent 1  At home, she doesn't really mask at all, you know, with immediate family that she's used to, but if we were to have friends round, if they ask questions and she's not comfortable with them, that's when she would do the silliness to try and get out of it. Or sometimes she doesn't always sort of join in… I think she sort of knows, you know, that they might ask a question, so she sort of keeps her distance. – Parent 5  And there are children who will use symbols comfortably at home, but are less comfortable in using them with peers because they don't want it to look any different. – SLT4 |

**Supplementary Materials: Interview topic guides**

We include here the topic guides developed to support the interviews with our participants. Note that the guides were tailored for either SLTs or parents. Prior to each interview commencing, we checked our participants understood the purpose of the study, how the data would be recorded and handled, and that they could request to stop at any time.

Topic Guide 1: Interviews with Speech and Language Professionals

***Part 1 – What is camouflaging in DLD, and what are its impacts?***

- As I’ve mentioned, we’re interested in whether children with DLD do things that might mask their difficulties, a process that we’re calling camouflaging. Based on our definition, do you think children with DLD camouflage?
- What does camouflaging “look like” in DLD? What behaviours do you see?
- How do you think camouflaging might affect identifying children’s language needs and the diagnostic process?
- How might it affect treatment or support?
- Might camouflaging benefit some children in some way? Are there any positives to camouflaging behaviour?

***Part 2 – Describing a specific case***

- In this next part of the interview, we’d like you to bring to mind a particular case you’ve worked with where camouflaging was going on. Preferably this would be a child with Developmental Language Disorder, or a profile very similar to this. Can you bring to mind a case of a child who camouflaged a lot?
- What did this child do that you would consider to be camouflaging? [Possible follow up: Was it successful – as in, did they disguise their language needs?]
- How did it affect the diagnosis and treatment of this child?
- How representative is this case of the sorts of camouflaging you see in your practice? Can you think of similar cases, or cases that looked a bit different?

***Part 3 – Factors associated with camouflaging***

- In your experience, are there any factors that are associated with camouflaging, such as gender, age, background or types of language need?
- So would you say there is there a particular profile of those who camouflage?
- To what extent do you think the camouflaging behaviours we’ve discussed today are specific to children with Developmental Language Disorder? Or do some behaviours cut across different diagnostic groups?

***Part 4 – Future research and developing measures***

- We’re coming to end of the interview now. One of the future aims of our research is to examine ways of identifying and measuring camouflaging in children with DLD. So could you tell me, what are the most common 2-3 camouflaging behaviours you see?
- If we were to create a questionnaire or observation measure for camouflaging in DLD, what do you think should be included?
- In the future, we hope to interview young people with DLD on this topic, to get their views and experiences with regards to camouflaging. Do you think children and adolescents with DLD are aware of their camouflaging?
- Do you have any advice for consulting young people with DLD? What might help us make the session as accessible for them?

Topic Guide 2: Interviews with Parents of children with DLD

**Part 1: Diagnosis**

- I’d like to start by asking about how your child’s diagnosis came about. When did you first notice that you child’s language was different?
- How did your child come to be diagnosed with DLD?
- How old was your son/daughter when they got the diagnosis?
- What sorts of difficulties does your child have?
- What were some of the things that made the diagnosis difficult? Was there agreement amongst family and teachers that there was a language problem?
- After the diagnosis, did support get put in place at school?

**Part 2: Remembering back to before diagnosis**

- I’d like to ask about before the diagnosis. Were there things that your son/daughter did that made the language needs harder for people to notice?
- Why do you think they did these things?
- Do you think that had an impact on the diagnosis? Either how long it took to be diagnosed, or anything else?
- Do you think the camouflaging impacted others’ perceptions of them? For example, what their teachers thought about them?

**Part 3: After diagnosis**

- Since this diagnosis, do they still do the behaviours you have described?
- Have they started to camouflage in other ways, do you think?
- Do you think the reasons for their camouflaging may have changed as they’ve gotten older?
- How successful was the camouflaging? Do people not realise they have misunderstood things?
- Has the camouflaging had an impact on any support they’ve received since their diagnosis? For example with TAs, or SLTs?
- Do you think camouflaging impacts their mental health or self esteem?
- Do you think camouflaging impacts their relationships?
- Are there certain scenarios where you notice your child camouflaging more?
- What are the top three camouflaging behaviours you think your child does?

**Part 4: Future research with young people**

- We’re interested in asking YP about this topic. What would need to take into account?

**Is there anything else you’d like our research team to know about this topic**
